# Chemometric investigation of light-shade effects on essential oil yield and morphology of Moroccan *Myrtus communis* L.

**DOI:** 10.1186/s40064-016-2749-5

**Published:** 2016-07-12

**Authors:** Mouhcine Fadil, Abdellah Farah, Bouchaib Ihssane, Taoufik Haloui, Sara Lebrazi, Badreddine Zghari, Saâd Rachiq

**Affiliations:** Laboratory of Functional Ecology and Environment, Faculty of Sciences and Techniques, Sidi Mohamed Ben Abdellah University, Fez, Morocco; Application Organic Chemistry Laboratory, Faculty of Sciences and Techniques, Sidi Mohamed Ben Abdellah University, Fez, Morocco; Laboratory of Microbial Biotechnology, Faculty of Sciences and Techniques, Sidi Mohamed Ben Abdellah University, Fez, Morocco; Laboratory of Applied Chemistry, Faculty of Sciences and Techniques, Sidi Mohamed Ben Abdellah University, Fez, Morocco

**Keywords:** Sun plants, Shade plants, Essential oil yield, Morphological parameters, Chemometrics methods

## Abstract

**Background:**

To investigate the effect of environmental factors such as light and shade on essential oil yield and morphological traits of Moroccan *Myrtus communis*, a chemometric study was conducted on 20 individuals growing under two contrasting light environments.

**Results:**

The study of individual’s parameters by principal component analysis has shown that essential oil yield, altitude, and leaves thickness were positively correlated between them and negatively correlated with plants height, leaves length and leaves width. Principal component analysis and hierarchical cluster analysis have also shown that the individuals of each sampling site were grouped separately. The one-way ANOVA test has confirmed the effect of light and shade on essential oil yield and morphological parameters by showing a statistically significant difference between them from the shaded side to the sunny one. Finally, the multiple linear model containing main, interaction and quadratic terms was chosen for the modeling of essential oil yield in terms of morphological parameters.

**Conclusions:**

Sun plants have a small height, small leaves length and width, but they are thicker and richer in essential oil than shade plants which have shown almost the opposite. The highlighted multiple linear model can be used to predict essential oil yield in the studied area.

## Background

Environmental factors such as light intensity, temperature, and water availability, have a major effect on the productivity of plants (Radušienė et al. 2012). Comparative studies on leaf characteristics of plants grown at high and low sunlight levels can give indications on leaves acclimatization to sun and shade environments, through adjustments that allow the greatest possible efficiency in the use of radiant energy (Givnish 1983). Moreover, plants of the same species belonging to different environmental conditions may show a significant change in their content of secondary metabolites (Szakiel et al. 2010).

*Myrtus communis* L. is an evergreen shrub belonging to the *Myrtaceae* family (Bruna et al. 2007; Snow et al. 2011). It is one of the important aromatic and medicinal species with high essential oil content in its leaves, flower and fruit glands (Aleksic et al. 2014). It’s a widely used spice that has many applications in the perfumery, food and pharmaceutical industries (Sumbul et al. 2011). It grows spontaneously in Morocco and is found in forest areas belonging to the thermo-Mediterranean series from the Atlantic coast to altitudes of 1100 m (Farah et al. 2006) under different conditions of light availability from full sunlight to canopy shading (Mendes et al. 2001).

The aim of this work was to evaluate the effect of light-shade natural stress on the growth and the essential oil yield of *Myrtus communis* plants growing in natural conditions in two shaded and exposed sites. The studied population was constituted by twenty individuals on whom we have recorded the values of the followed characters: Essential oil yield, morphological and geographical parameters. In this way, we began firstly by detecting correlations between parameters and searching groups of similar individuals by principal component analysis (PCA) and hierarchical cluster analysis (HCA). Later, we have confirmed the light-shade effect on each of parameters by one-way analysis of variance. Finally, we have looked for a model linking the essential oil yield with morphological parameters by multiple linear regression (MLR).

## Methods

### Field site and plant material

**Fig. 1:**
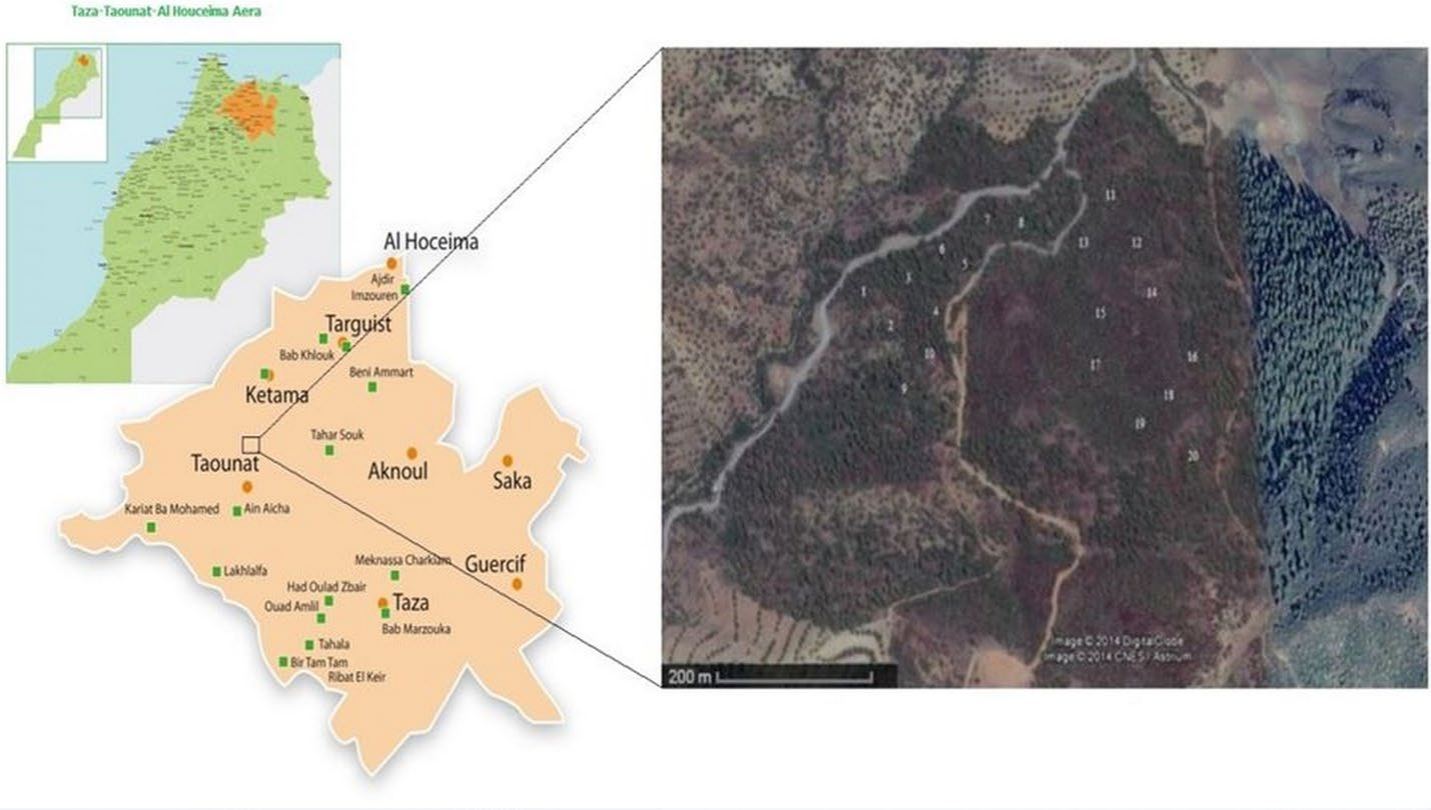
Satellite image of *Myrtus communis* sampling site and its localization in the map of Taza-Taounat-Al Houceima area

The study was conducted on a population of *Myrtus communis* in “Ifran” forest located on the shores of the “Sahla” dam at 22 km from Taounate city in Taza-Taounat-Al Houceima Aera (Morocco) (latitude 34°35′02.1″N; longitude 4°38′37.7″W) (Fig. 1). The choice of the studied individuals was based on their exposure to sunlight. Thus, individuals from 1 to 10 were collected from the shaded site (shade plants) while individuals from 11 to 20 were collected from the exposed site (sun plants). *Myrtus communis* plants were harvested at the same time, in the middle of October (Ripen fruits Period) which is the best period for better exploiting the myrtle essences (Pereira et al. 2009; Fadil et al. 2015). The botanical identification of species was carried out by the botanists of the National Institute of Medicinal and Aromatic Plants (NIMAP). The authenticated voucher specimen was deposited in the institute Herbarium.

### Extraction material

The Clevenger apparatus was used for hydrodistillation (Clevenger 1928). For each individual, 100 g of *Myrtus communis* leaves and water were placed, in determined proportions, in a one-liter capacity glass flask. The mixture was heated to boiling temperature for 3 h and the liberated steams crossed up the column and passed out of the condenser in a liquid state. The obtained essential oil was dried over anhydrous sodium sulfate and was stored in the refrigerator at 4 °C in dark glass bottles until use.

### Studied parameters

The studied population was constituted by twenty individuals on whom we have recorded the values of several parameters as follows:

Metabolic parameter:

- The essential oil yield determined by hydrodistillation. Retained value is the average of three extractions on the same individual.

Morphological traits:

- The height of each individual was measured from the ground level to the highest leaf at the top;
- The leaves length expressed by the average of thirty leaves lengths taken from the third considered section after the division of the aerial part of every individual in four equal sections extending from the ground level to the highest leaf at the top;
- The leaves width expressed by the average of thirty leaves widths taken from the third considered section after the division of the aerial part of every individual in four equal sections extending from the ground level to the highest leaf at the top;
- The whole-leaf thickness expressed by the average of thirty leaves thickness taken from the third considered section after the division of the aerial part of every individual in four equal sections extending from the ground level to tip of the top leaf. Leaf thickness was measured by using a digital Vernier Caliper.

Geographical parameter:

- The altitude of each individual measured by a GPS localization instrument.

### PCA, HCA and one-way ANOVA

The loading plot tool in PCA was used to show the relationship between variables and how significant each variable was for each principal component. As well, the score plot was used to identify individuals’ groupings showing a similarity. For better visualization of individuals’ grouping, we have used the hierarchical cluster analysis (HCA) technique which is a graphical representation of a distance matrix. The result of this classification tool is a graph which shows the class-inclusive relations between clusters and the value of the clustering criterion associated with each (Bridges 1966). To confirm the effect of sunlight exposition on each of studied parameters, a one-way ANOVA test was used. This is a statistical tool used to test the claim that two or more population means are equal. The Unscrambler software (version 9.7) for chemometric analysis was used to perform PCA, while HCA, one-way ANOVA were carried out using Statgraphics Centurion XVI Software.

### Multiple linear regression

To find the model linking essential oil yield to morphological parameters, a multiple linear regression (MLR) was used. This is a statistical technique which involves finding a linear relationship between a dependent variable Y_i_ (j = 1, 2, 3…, q) and more than one independent variable X_j_ (j = 1, 2, 3,…, p). For this step of the study, three models were tested. The first was a model which contains only main terms; the second was a model which contains both main and interaction terms while the third was a model including all the main, interaction and quadratic terms. The choice of the model was based on the results of the variance analysis and those of the coefficient of determination R^2^ and adjusted coefficient of determination R^2^a. The statistical significance of the model coefficients was determined by using the *t student* test. The model coefficients were considered significant for values of *p* value <0.05. SPSS Software (version 20) was used to perform multiple linear regression.

## Results

### Recorded values for the studied parameters

The recorded values for the six studied parameters are summarized in Table 1. The results have indicated that essential oil yield was ranged from 0.48 to 0.93 % in the shaded site. While, in the exposed site, we have noted a small increase in essential oil yield which varied between 0.88 and 1.06 %. Altitudes in the shaded site were between 452 and 470 m while this parameter was between 467 and 485 m in the exposed site. Otherwise, in shaded site morphological parameters were comprised between the values 146–200 cm for the individual height, 3.25–4.5 cm for the leaves length, 1.01–1.58 cm for the leaves width and 189–215 µm for the leaves thickness. Concerning exposed site, morphological parameters were comprised between the values 67–130 cm for the individual height, 2.81–4.05 cm for the leaves length, 0.65–1.08 cm for the leaves width and 375–412 µm for the leaves thickness.

**Table 1:** Essential oil yield, morphological traits, and altitude recorded for twenty studied individuals

Sampling site	Individuals	Essential oil yield^a^ (%)	Altitude (m)	Height (cm)	Leaves length^b^ (cm)	Leaves width^b^ (cm)	Leaves thickness^b^ (µm)
Shaded	Ind 1	0.48 ± 0.033	455	175	3.6 ± 0.081	1.21 ± 0.074	189 ± 0.078
Sahded	Ind 2	0.48 ± 0.045	454	178	3.74 ± 0.052	1.26 ± 0.110	192 ± 0.066
Sahded	Ind 3	0.76 ± 0.039	462	156	4.1 ± 0.067	1.58 ± 0.065	195 ± 0.092
Sahded	Ind 4	0.93 ± 0.051	470	146	4.5 ± 0.082	1.38 ± 0.0.09	198 ± 0.053
Sahded	Ind 5	0.8 ± 0.011	465	162	3.78 ± 0.063	1.5 ± 0.037	200 ± 0.058
Sahded	Ind 6	0.54 ± 0.063	452	180	3.46 ± 0.045	1.1 ± 0.052	202 ± 0.039
Sahded	Ind 7	0.57 ± 0.022	455	170	4.17 ± 0.056	1.42 ± 0.081	206 ± 0.052
Sahded	Ind 8	0.7 ± 0.041	465	160	3.59 ± 0.091	1.14 ± 0.111	209 ± 0.064
Sahded	Ind 9	0.72 ± 0.017	464	200	4.06 ± 0.064	1.22 ± 0.078	212 ± 0.073
Sahded	Ind 10	0.65 ± 0.009	466	150	3.25 ± 0.049	1.01 ± 0.098	215 ± 0.059
Exposed	Ind 11	1.06 ± 0.035	482	90	4.05 ± 0.10	1.08 ± 0.089	369 ± 0.081
Exposed	Ind 12	0.96 ± 0.055	485	105	3.08 ± 0.054	0.91 ± 0.056	375 ± 0.084
Exposed	Ind 13	0.95 ± 0.024	467	95	3.74 ± 0.074	1.06 ± 0.064	386 ± 094
Exposed	Ind 14	0.99 ± 0.016	472	67	3.28 ± 0.068	0.9 ± 0.071	392 ± 0.083
Exposed	Ind 15	0.96 ± 0.043	475	92	3.31 ± 0.043	0.89 ± 0.059	395 ± 0.113
Exposed	Ind 16	0.88 ± 0.049	478	100	2.81 ± 0.051	0.65 ± 0.091	398 ± 0.086
Exposed	Ind 17	0.88 ± 0.022	474	85	3.13 ± 0.072	0.97 ± 0.032	401 ± 0.069
Exposed	Ind 18	1.02 ± 0.062	475	130	3.35 ± 0.084	1.2 ± 0.088	404 ± 0.0117
Exposed	Ind 19	0.98 ± 0.051	482	110	3.08 ± 0.046	0.91 ± 0.061	408 ± 0.057
Exposed	Ind 20	0.99 ± 0.019	480	70	3.5 ± 0.095	1.08 ± 0.048	412 ± 0.078

^a^Each value represents the mean of 3 replicates with standard error

^b^Each value represents the mean of 30 replicates with standard error

### Principal component analysis

#### Explained variability

To decide about the number of retained components, we have adopted the Kaiser criterion which says that during a standardized ACP, we must keep components whose eigenvalues are higher than 1 (Jackson 1991). The results (Table 2) have shown that the first two components satisfied this criterion and they can be considered to explain data variability. Table 2 has also displayed the percentages of explained variability by each component and the cumulated percentages. The first component which explains 72.22 % of the data variability was mostly contributed by essential oil yield, altitude, height and leaves thickness, while the second which explains 18.87 % was mostly contributed by leaves length and leaves width. So, we were satisfied by retaining these two components explaining 91.09 % of the total data variability.

**Table 2:** Coordinates of parameters on each component, eigenvalues of components, explained variability by each of them and cumulated percentages of explained variability

Component number	PC1	PC2	PC3	PC4	PC5	PC6
Essential oil yield	−0.835	0.519	0.084	0.020	0.074	0.144
Altitude	−0.900	0.271	0.311	−0.040	−0.096	−0.092
Height	0.920	−0.167	0.308	−0.021	0.173	−0.004
Leaves length	0.667	0.693	−0.107	−0.244	0.034	−0.044
Leaves width	0.793	0.524	0.009	0.309	−0.031	−0.030
Leaves thickness	−0.963	0.048	−0.136	0.085	0.190	−0.097
Eigenvalues	4.33	1.13	0.24	0.16	0.09	0.05
Percentages of explained variability (%)	72.22	18.87	3.93	2.68	1.51	0.8
Cumulated percentages (%)	72.22	91.09	95.02	97.69	99.2	100

#### Parameters study

**Fig. 2:**
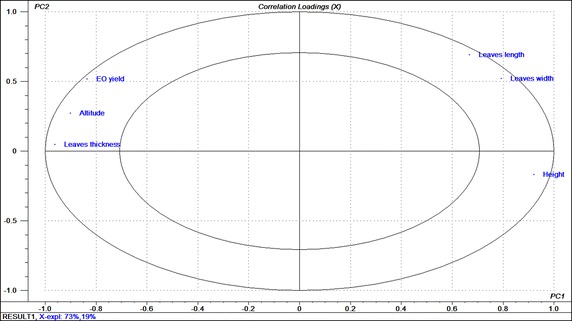
*Loading plot* which shows that essential oil yield, leaves thickness, and altitude were positively correlated between them and negatively with other morphological traits (plants height, leaves length and width); PC1and PC2 are the first two principal components

The Loading plot (Fig. 2) has revealed the existence of some correlations between studied parameters, in particular, those between essential oil yield, leaves thickness, and altitude which were positively correlated and the morphological parameters which were also positively correlated between them and negatively correlated with the first three parameters. These results mean that the more the altitude increases the shorter the individuals become and the less high and wide leaves get. However, they also become richer in essential oils and their leaves get thicker.

#### Individuals study

**Fig. 3:**
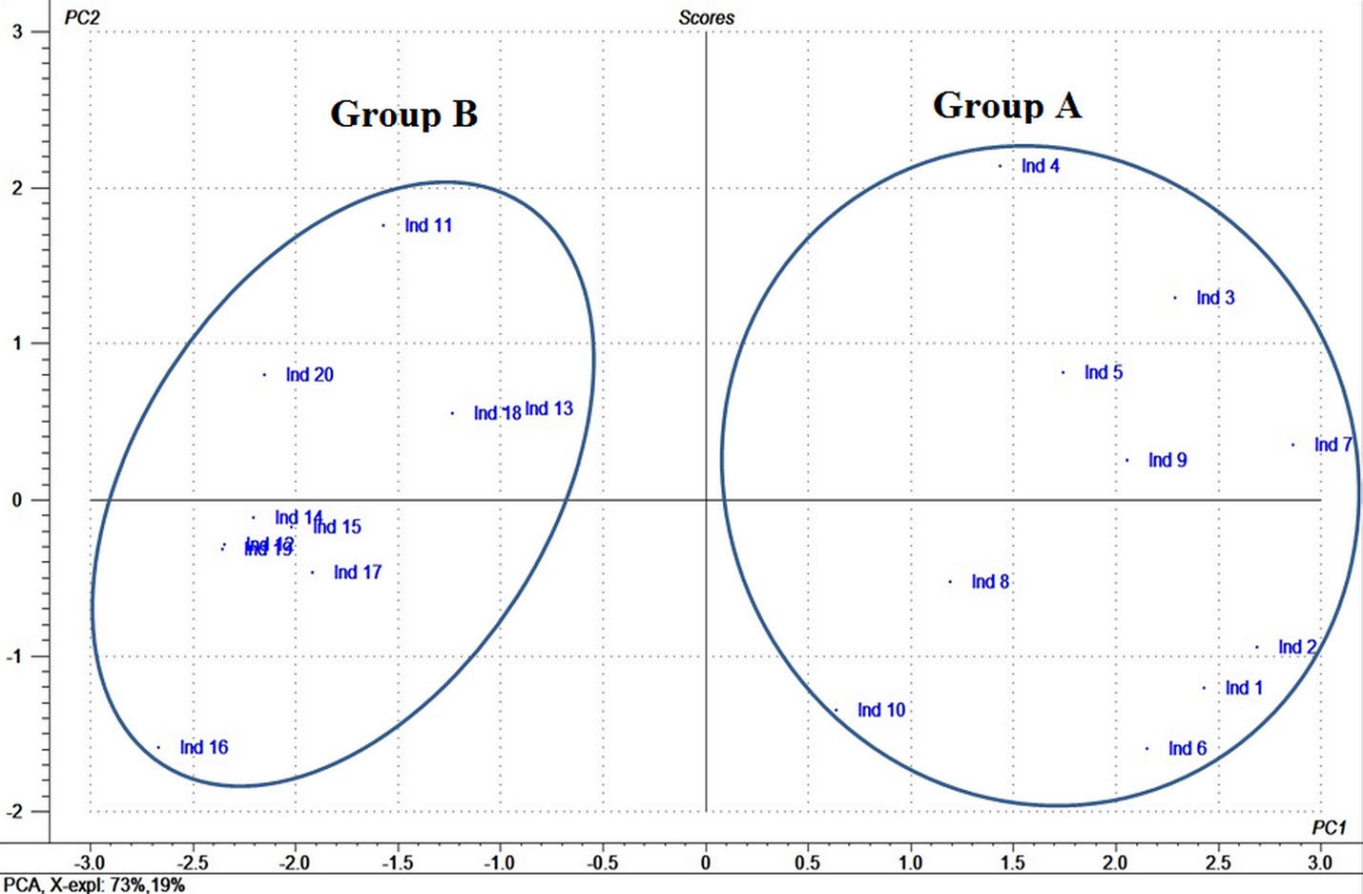
Graph of individuals’ distribution (*score plot*) showing two groups of individuals classified according to the studied parameters. PC1and PC2 are the first two principal components

The score plot (Fig. 3) has illustrated two groupings of individuals each one contains similar individuals according to the studied parameters. Consequently, we have observed that the individuals 1–10 (shade plants) formed a group on the right of the first component (group A), while the individuals 11–20 (sun plants) composed a group on the left (group B).

#### Parameters and individuals bi-plot

**Fig. 4:**
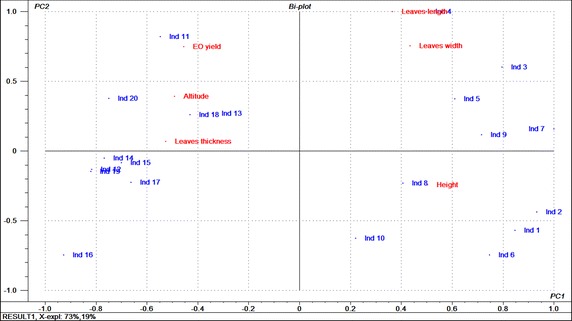
Bi-plot got by superposing score and loading plots showing the distribution of individuals and parameters. PC1and PC2 are the first two principal components

By superposing the score and loading plots, we have got the bi-plot (Fig. 4). According to this graph, we notice that shade plants were higher, have longer and wider leaves, but they were thinner and poorer in essential oil. Conversely, sun plants were shorter, have less long and wide leaves, but they are thicker and richer in essential oil.

### Hierarchical cluster analysis

**Fig. 5:**
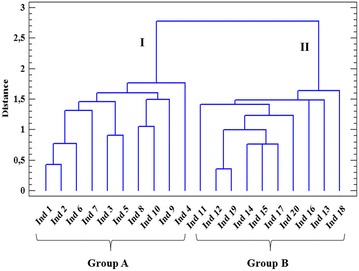
Hierarchical cluster analysis of *Myrtus communis* individual’s based on the studied parameters. The between-group method based on the Euclidian distance was used; *group A*: shade plants, *group B*: sun plants

Aiming at a better visualization of the investigated population classification according to altitude, essential oil yield and morphological parameters, a hierarchical cluster analysis (HCA) was carried out (Fig. 5). As a confirmation of PCA score plots, the individuals were regrouped in two main clusters. Cluster I represents shade plants (Individuals from 1 to 10 belonging to group A in the PCA analysis) and Cluster II represents sun plants (Individuals from 11 to 20 belonging to group B in the PCA analysis).

PCA and HCA results have pushed us to suppose that essential oil yield and morphological parameters are affected by the difference in environmental conditions such as sun exposure.

### One-way ANOVA test

The results of Table 1 have shown a slight increase in essential oil yield observed in sun plants compared to shaded ones. Also, for the three morphological parameters height, leaves length, and leaves width, shade plants were characterized by higher values than those found within sun ones. However, for the fourth morphological parameters (leaves thickness), we observed the opposite. To confirm the effect of the sun exposure on the essential oil yield and morphological parameters, a one-way ANOVA was conducted by considering the sampling site as a variable (Table 3).

**Table 3:** Means and standard deviations with the F_calculated_ obtained for the one-way ANOVA test carried out on the values of essential oil yield and morphological parameters by considering the sampling site as a factor

	Shades site n = 10	Exposed site n = 10	F_Ratio_
Essential oil yield (%)	0.66 ± 0.044	0.96 ± 0.018	37.7*
Height (cm)	167.7 ± 4.76	94.4 ± 5.88	88.14*
Leaves length (cm)	3.83 ± 0.11	3.33 ± 0.11	8.93*
Leaves width (cm)	1.28 ± 0.053	0.96 ± 0.04	17.82*
Leaves thickness (µm)	201.8 ± 2.59	394 ± 4.39	1378.62*

* Significant at p < 0.001 probability

All of the studied parameters have shown a significant change regarding the studied factor (Table 3). This means that there is a statistically significant change in essential oil yield and morphological parameters by passing from shaded to exposed sites. These results confirm those obtained by PCA and HCA showing that the exposure to the sun had a direct influence on studied parameters by increasing essential oil yield and leave thickness and reducing plants height, leaves length and leaves width. Opposite results were confirmed for shaded plants.

#### Modeling of essential oil yield in terms of morphological parameters

From the precedents results, we have detected several correlations between essential oil yield and morphological parameters. To get more information about the essential oil yield in individuals belonging to the studied location, we sought to model it by a multiple model including morphological parameters as model coefficients. Before proceeding to the modeling, the values of each parameter were centered. This step allows the standardization of measurement units.

#### Variance analysis and model choice

The main effect of regression was statistically significant for the three tested models (*p* value <0.05) (Table 4). Obviously, the calculation of *F value* (F_calculated_) has shown that it was higher than the theoretical *F*-*value* (F_theoretical_) for the same degree of freedom at 95 % confidence level. Moreover, the coefficient of determination R^2^ and the adjusted coefficient of determination R^2^a of the third model have the highest values compared with the obtained values for the two first models (98.8 and 95.44 %, respectively). Therefore, we have chosen this model containing main, interaction and quadratic terms in the essential oil prediction.

**Table 4:** F_calculated_, F_theoretical_, *p* values, coefficients of determination R^2^ and adjusted coefficient of determination R^2^a related to the three tested models

	Model with main terms only	Model with main and interaction terms	Model with main, interaction and quadratic terms
F_calculated_	15.65***	7.26**	29.42***
F_theoritical_	3.05	3.13	4.63
*p* value	0	0.0032	0.0007
R^2^ (%)	80.67	88.97	98.8
R^2^a (%)	75.51	76.72	95.44

**, ***: Significant at p < 0.01 and p < 0.001 probability, respectively

**Fig. 6:**
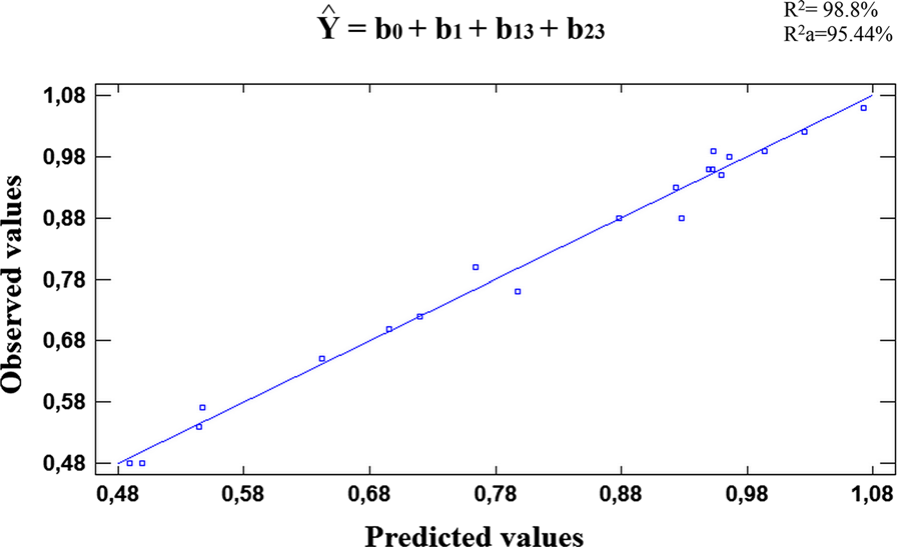
Curve of observed values as a function of predicted ones obtained for the third tested model containing all of the main terms, the interaction terms, and quadratic terms

The obtained R^2^ and R^2^a have given a good agreement between the experimental and the predicted values of the adapted model. These results were confirmed by those obtained in the graph of correlation showing a linear curve for the observed values in terms of the predicted ones (Fig. 6).

#### Coefficients estimation and mathematical model

Coefficients estimations, statistical *t student* values, and the observed probability (*p value*) for each of the coefficients are summarized in Table 5. The results have shown that the factors b_0_, b_1_, b_13_, b_23_ have a statistically significant effect (*p* value <0.05). As a result, these coefficients were included in the fitted model.

**Table 5:** Estimated regression coefficients for the model containing main, interaction and quadratic terms

Coefficients	Coefficient	Estimation	Standard error	*t* student	*p* values
Constant	b_0_	1.003	0.075	13.288	<0.001***
Height	b_1_	−0.004	0.001	−5.163	0.003**
Leaves length	b_2_	0.131	0.096	1.367	0.230
Leaves width	b_3_	−0.257	0.209	−1.229	0.274
Leaves thickness	b_4_	0.000	0.000	0.118	0.911
Height*Leaves length	b_12_	0.002	0.004	0.494	0.642
Height*Leaves width	b_13_	−0.026	0.007	−3.710	0.013*
Height*Leaves thickness	b_14_	0.000	0.000	2.542	0.052
Leaves length*Leaves width	b_23_	−2.688	0.952	−2.824	0.036*
Leaves length*Leaves thickness	b_24_	−0.003	0.002	−1.259	0.264
Leaves width*Leaves thickness	b_34_	−0.006	0.005	−1.241	0.270
Height*Height	b_11_	0.000	0.000	2.457	0.058
Leaves length*Leaves length	b_22_	0.210	0.223	0.940	0.390
Leaves width*Leaves width	b_33_	2.859	1.359	2.105	0.089
Leaves thickness*Leaves thickness	b_44_	0.000	0.000	−0.215	0.838

*, **, ***: Significant at p < 0.05, p < 0.01 and p < 0.001 probability, respectively

The mathematical model used for modeling the essential oil yield as a function of morphological parameters is represented as shown in Eq. 1:

1 $$(Essential\,oil\,yield (\% ))_{Estimated} = 1.003 - 0.004*\left( {Height} \right) - 0.026*\left( {Height*Leaves\,width} \right) - 2.688*\left( {Leaves\,length*Leaves\,width} \right)$$

This model can be used to estimate the essential oil yield of the studied area and can be a useful tool in the choice of individuals to operate.

## Discussion

As it was confirmed by the precedent results, the sun exposure had a negative effect on the morphological characteristics of the individuals except leaves thickness which showed an increase. However, in terms of essential oil yield, the results revealed a remarkable increase in sun plants compared with shade ones (Table 1). These observations were proved by the PCA study which showed the existence of a positive correlation between essential oil yield, leaves thickness and altitude. Besides, the one-way ANOVA has confirmed the existence of a significant difference between essential oil yield and morphological parameters found within both populations. This difference was attributed to light-shade conditions present in both studied sites.

Considered as a stressor factor (Rzigui et al. 2015), light is one of the most diverse factors that affect the plants growth and development (Gomez-Aparicio et al. 2006). Furthermore, under natural conditions, a stress due to strong sunlight is rarely an isolated phenomenon; It is often associated with an increase in temperature (Braun et al. 2002). In addition, there is a drought stress caused by water deficit, usually accompanied by high temperatures and solar radiation (Xu et al. 2014). Indeed, if the leaf receives a radiation, its temperature increases and increases the transpiration in leaves (Jones 2014).

### Essential oil yield

The observed difference in essential oil yield can be explained by sunlight exposition (Foyer et al. 1994). First, light acts on the biosynthetic pathways activation of essential oils, which are dependent on the carbon chain obtained by photosynthetic processes. This latter is generally higher in light environments (Schuh et al. 1997). Second, several studies have reported that the advanced generation of ROS (reactive oxygen species) in plants due to disruption of cellular homeostasis is a result of environmental stresses such as light radiation (Sharma et al. 2012). In fact, there will be an increase in ROS production as a result of oxidative stress caused by exposure to abiotic factors such as light and temperature (Shohael et al. 2006). A lot of troubles such as peroxidation of lipids, oxidation of proteins, damage of nucleic acids, enzyme inhibition, activation of programmed cell death (PCD) pathway and ultimately leading to the death of the cells, can affect plants by the advanced production of ROS during environmental stresses (Sharma et al. 2012). To find a way out, plants contain a number of enzymatic and non-enzymatic mechanisms that ensure their protection against increased accumulation of ROS (Inzé and Van Montagu 1995). However, activation of antioxidant enzymatic and non-enzymatic is probably not sufficient to scavenge ROS. Therefore, it’s possible that other mechanisms may be induced for detoxification, more specifically the increased production of essential oils (Gniazdowska et al. 2015). These molecules play a major role in the plant adaptation to the changing environmental conditions and in confronting stress constraints. Surely, essential oils could stabilize cell membranes by binding either the lipid bilayer, or the protein membrane interface, or the photosystem II subunits (Sharkey and Loreto 1993). This could provide a general protection against environmental constraints (Sharkey and Loreto 1993). Besides this, essential oils production can also be positively affected by drought stress thanks to the water deficit resulting from temperature and solar radiation. Indeed, it has been reported that essential oil yield activity is strongly influenced by temperature (Jochum et al. 2007). Water deficits appear to have a role in a higher density of glandular hairs and a greater essential oils contained in leaves produced under water stress conditions (Gershenzon et al. 1978). This is another example of a physiological mechanism for the increased accumulation of essential oils under stress. The increased amounts of essential oils appears to be the result of changes in growth and development, rather than the direct influence of stress on secondary metabolism (Timmermann et al. 2013).

Many studies have confirmed the relationship between environmental factors such as exposure to strong light and the production of secondary metabolites (Stocker-Worgotter 2001). When intense sunlight seems to be directly related to an increase in the production of volatiles in some species such as *Anethum graveolens*, *Artemisia dracunculus,* and *Ocimumbasilicum* (Figueiredo et al. 2008), a reduction of essential oil contained under low solar radiation levels has been reported in Japanese mint (Dutta 1971), *Mentha cordifolia Opiz* (Cantoria and Cuevas-Gacutan 1974) and basil (Chang et al. 2008).

### Morphological parameters

In our case, microclimate change due to light-shade conditions has caused a clear difference between *Myrtus communis* individuals belonging to the studied sites. Morphological parameters were higher under shaded conditions and lower under sunlight exposure ones except leaves thickness, which showed contrary results. As in the case of essential oils yield, this difference is mainly due to the individual’s adaptation to the abiotic factors such as light intensity, temperature, and water deficit.

Generally, it’s expected that plant growth will increase with increasing irradiance (Duan et al. 2005). However, a growth decrease at high light conditions is also observed (Korner 1991). Species may differ in their relative growth rate (RGR) as a result of a given radiation (Poorter 1999). Plant growth analysis decomposes RGR into net assimilation rate (NAR, rate of dry matter production per unit leaf area) and leaf area ratio (LAR, leaf area per unit total plant mass) (Blum 2011). NAR is determined primarily by the ratio of carbon gained through photosynthesis and carbon lost through respiration. LAR depends on the proportion of biomass allocated to leaves relative to total plant mass (leaf mass ratio, LMR) and how much leaf area a plant develops per unit leaf biomass (specific leaf area, SLA) (James and Drenovsky 2007). SLA is a major parameter of growth rate because it’s proportional to the capturing light area per unit of previously captured mass (Xu et al. 2009).

Shade plants have a higher biomass allocation to leaves and a higher leaf area per unit leaf mass (SLA), resulting in a higher leaf area per unit plant mass (LAR) (Popma and Bongers 1988; Osunkoya et al. 1994). In shaded conditions, plants generally present an increase in the investment in leaf area in order to enhance light interception (Pearcy and Sims 1994; Niinemets et al. 1999). Furthermore, shade plants have a higher stem length per unit stem biomass which increases the height growth in a way that facilitates escape from the low-light environment (Sasaki and Mori 1981). This process is in total agreement with our results which report that shade plants were higher than sun ones. By contrast, and because they are subjected to large evaporation, the sun leaves usually take an avoidance strategy by reducing water loss and increasing carbon gain through the decrease of leaf size and an increase of leaf thickness (Mendes et al. 2001). Consequently, there is a decrease in SLA (Xu et al. 2009) which is necessary to adjust the plant transpiring surface area in response to increased light (Shipley 1995).

Accordingly, there is the notion that plants have a remarkable ability to adapt the growth of their organs to environmental conditions. In a low light environment plants should allocate biomass to light-capturing tissue and minimize carbon losses, but in the high light environment, plants invest more in root mass in order to compensate for higher transpiration losses by water uptake (King 1994; Valio 2001). In the second case, less biomass can be invested in leaf material, which strongly reduces photosynthetic gain and potential growth rate (Korner 1991). These results are completely in agreement with ours. Similar results have been obtained for leaves of Portugal *Myrtus communis* (Mendes et al. 2001), *Corylus avellana* (Catoni et al. 2015) and *Vitis vinifera* L. (Pollastrini et al. 2011) which have shown the same response to different light levels.

## Conclusions

The results of the performed PCA, HCA and one-way ANOVA have confirmed that there is a close link between light-shade conditions and essential oil yield and morphological parameters. Sun exposure involves a light and thermal stress which acts on the morphological and physiological mechanisms in the concerned individuals; this process promotes the production of secondary metabolites such as essential oil yield which is one of the various mechanisms used by plants to protect the photosynthetic apparatus against damage from the accumulation of excessive light and thermal energy. Concerning morphological parameters, an important adjustment was observed in order to promote more efficient carbon assimilation in sun plants. In contrast, shade ones improved their height and surface area for light capture. These results can be an outstanding asset in favor of industrial exploiters of *Myrtus communis* essential oils. Indeed, since the adaptation to light-shade is primarily marked by a highest production of essential oils, we recommend that exploitation must be oriented to areas submitted to intense sunlight exposition. In parallel, the use of the established model by multiple linear regression can give an important information about essential oil yield prediction in the studied area.
